# Swimming Kinematics and Hydrodynamics of a Subtropical Sea Angel

**DOI:** 10.1093/icb/icag160

**Published:** 2026-08-29

**Authors:** Abdullah Aldaddi, Evan Williams, Ferhat Karakas, Amy Maas, David Murphy

**Affiliations:** Department of Mechanical and Aerospace Engineering, University of South Florida, Tampa, FL33620, USA; Department of Mechanical and Aerospace Engineering, University of South Florida, Tampa, FL33620, USA; Department of Mechanical and Aerospace Engineering, University of South Florida, Tampa, FL33620, USA; Bermuda Institute of Ocean Sciences, Arizona State University, 17 Biological Station, St Georges, GE 01, Bermuda; Department of Mechanical and Aerospace Engineering, University of South Florida, Tampa, FL33620, USA

## Abstract

Sea angels (gymnosomatous pteropods) are small zooplanktonic shell-less marine snails inhabiting the meso- and epipelagic zones. They swim in an intermediate Reynolds number regime using highly flexible, wing-like parapodia in order to capture prey, avoid predators, and perform diel vertical migration. However, the kinematics and fluid dynamics of gymnosome swimming are not well understood, particularly for species residing in low-viscosity, subtropical waters. Here we use high-speed stereophotogrammetry and dual brightfield particle image velocimetry (PIV) systems to investigate the swimming of the rare subtropical species *Pneumoderma atlantica*, captured off the coast of Bermuda. In particular, we quantify wing kinematics for hovering and slow upwards swimming and compare our results with morphologically similar temperate and polar species, which can be up to twice as large and swim in water up to twice as viscous. Like tiny insects flying in a similar regime, the chordwise Reynolds number appears to be inversely related to the wing angle of attack and stroke plane. Thus both the small, warm-water and the large polar gymnosomes seem to use their wings more like paddles to generate upward forces while the temperate species seems to use its parapodia more like wings to generate lift. Further, we provide the first flow measurements of a swimming gymnosome, these at somewhat higher swimming speeds, which show that gymnosomes employ an unsteady flow interaction between the wings and body (similar to the clap-and-fling mechanism) twice during each stroke cycle which likely generates additional lift. These findings provide insight into how similar locomotion modes may be adapted to different viscosities and into the widespread use of lift-generating, clap-and-fling-like mechanisms among marine snails.

## Introduction

Gymnosomatous pteropods, or sea angels, are holoplanktonic marine zooplankton that inhabit the mesopelagic and epipelagic layers (Lalli and Gilmer 1989). Their bodies are streamlined and range in length from less than 2 mm up to approximately 70 mm for the temperate species *Clione limacina* (Conover and Lalli 1972; Lalli and Gilmer 1989). These sea snails lose their shells during development and the foot has been modified into a pair of flexible wing-like appendages called “parapodia” (Satterlie et al. 1985; Lalli and Gilmer 1989). Gymnosomes have a global distribution, though their occurrence is low at the equator and greater at higher latitudes (Burridge et al. 2017). Though gymnosomes are numerically less common than their main prey, the shelled (thecosomatous) pteropods, they are still an important part of the food web (Lalli and Gilmer 1989; Gilmer 1990; Burridge et al. 2017) and are themselves consumed by larger animals like fish and baleen whales (Lalli 1970; Lalli and Gilmer 1989). However, gymnosomes are not well studied as their soft bodies are easily damaged in net tows (Lalli 1970), and field observations are limited.

Swimming is an important but poorly understood behavior for gymnosomes. Gymnosomes are slightly negatively buoyant and must continuously flap their parapodia to avoid sinking. Further, swimming is important to find mates, capture prey (Seibel et al. 2007), avoid predators, and to perform diel vertical migration (DVM). DVM is a common behavior among marine zooplankton in which they ascend to the upper water layers during the night to feed and sink back to depth during the day to avoid visual predators (Hays 2003). For example, *Pneumoderma atlantica*, the subject of this study, is thought to vertically migrate approximately 100 m (Karakas et al. 2020b). As another example, the polar species *C. antarctica* was found to be more than ten times as common in the top 2 m during the night as compared to during the day owing to DVM patterns (Flores et al. 2011).

Swimming by various gymnosome species has been studied previously. Satterlie et al. (1985) first investigated gymnosome swimming in the species *C. limacina* using high-speed filming. These researchers found that the wingtips trace a “figure-of-eight” trajectory, pointed out the similarities between gymnosome swimming and insect flight, and suggested that *C. limacina* uses a variant of the “clap-and-fling” (or Weis-Fogh) mechanism to generate lift (Weis-Fogh 1973). In this mechanism, the insect wings closely approach each other at the end of a half-stroke (the clap phase) and subsequently rotate or peel apart from each other about their trailing edges (the fling phase) as they begin to separate. This motion generates a V-shaped gap containing a low-pressure region into which air flows, thus generating enhanced leading edges vortices on the wings and increasing lift (Cooter and Baker 1977; Dickinson et al. 1999; Williams et al. 2025). In gymnosomes, the highly flexible wings lay close to the body at the end of each half-stroke and peel away from the body with the anterior (i.e., leading) edge moving before the trailing edge, a motion that likely creates a low-pressure region between the wing and the body (Satterlie et al. 1985). The analytical model of gymnosome swimming developed by Childress and Dudley (2004) also suggested the importance of wing-body interactions for lift generation.

Borrell et al. (2005) then measured the three-dimensional kinematics of slowly ascending *Clione antarctica*. These authors found that the parapodia angle of attack increased with swimming speed (in the range of 60°–80°), suggesting that they primarily generate forces by using their parapodia as paddles instead of wings, though the authors also point out that unsteady mechanisms including wing-body interactions may also be important. Szymik and Satterlie (2011, 2017) examined the 3D wing and body kinematics and the internal hemocoel fluid circulation of slow- and fast-swimming *C. limacina*. This species also flaps its parapodia in a “figure-of-eight” trajectory in both swimming modes. However, in fast swimming, the animal increases its angle of attack and pulls its wings closer to the body at the end of each half-stroke, such that the wingtips overlap or nearly touch. These researchers also suggest that wing-body interactions (in the form of flow being squeezed out from between the wing and body at the end of each half-stroke) may be important for lift production. Finally, Karakas et al. (2020a) used low magnification, high-speed recordings to measure the three-dimensional swimming trajectories of various warm-water pelagic snails, including *P. atlantica*. For two individuals in the size range of 11.5–13.1 mm beating their wings at 3.5–4.5 Hz, these gymnosomes often swam upwards in a spiral at speeds of 11–34 mm s^−1^, though hovering was also observed. As also noted by other researchers (Satterlie et al. 1985; Borrell et al. 2005; Szymik and Satterlie 2011), these animals pitch forwards and backwards with each half-wing stroke and swim in the intermediate Reynolds number range of several hundred, a regime in which both viscous and inertial effects are important.

Though several researchers have investigated gymnosome swimming, only kinematics and swimming speed measurements have been conducted to date. Flow measurements of gymnosomes have not yet been conducted but would be valuable in ascertaining the role of lift versus drag and of wing-body interactions in gymnosome swimming. Further, most research on gymnosomes to date has focused on temperate and polar species swimming in cold water (Satterlie et al. 1985; Borrell et al. 2005; Szymik and Satterlie 2011), with little focus on the rarer warm water species (Karakas et al. 2020b). This difference is important because water viscosity is known to vary by a factor of almost two from tropical to polar waters. The resulting difference in the Reynolds number would thus affect force generation and the formation and dissipation of vortices (Borrell et al. 2005; Szymik and Satterlie 2011; Mohaghar et al. 2019; Karakas et al. 2020a). Here we investigate the wing and body kinematics of the subtropical gymnosome *P. atlantica* during hovering and forward swimming and provide flow measurements around this species for forward swimming. We also compare our wing kinematics data with those of other gymnosomes found at different latitudes in order to examine the possible effects of water viscosity and Reynolds number on the swimming of these species.

## Materials and methods

### Species and environment

Zooplankton were captured offshore of Bermuda during nighttime cruises using a Reeve net with 150 µm mesh size and a specialized 20 l cod end. The animals were kept in collected seawater and transported back to the Bermuda Institute of Ocean Sciences (BIOS) to perform experiments in a temperature-controlled chamber. A stereomicroscope was used to sort and identify the pteropods, which were stored in filtered seawater at an *in situ* temperature of 21^o^C. Experiments were completed within 36 h, owing to the fact that the pteropods did not live more than several days after capture. One gymnosome, collected in May 2017, was used to measure the 3D wing and body kinematics while two individuals collected in May 2019 were used to measure flow fields. Low numbers of gymnosome pteropods were available because these animals are rare, especially at low latitudes (Burridge et al. 2017), and possibly because these animals are fast swimming and may be able to avoid sampling nets (Lalli and Gilmer 1989).

### 3-Dimensional kinematics setup

**Fig. 1:**
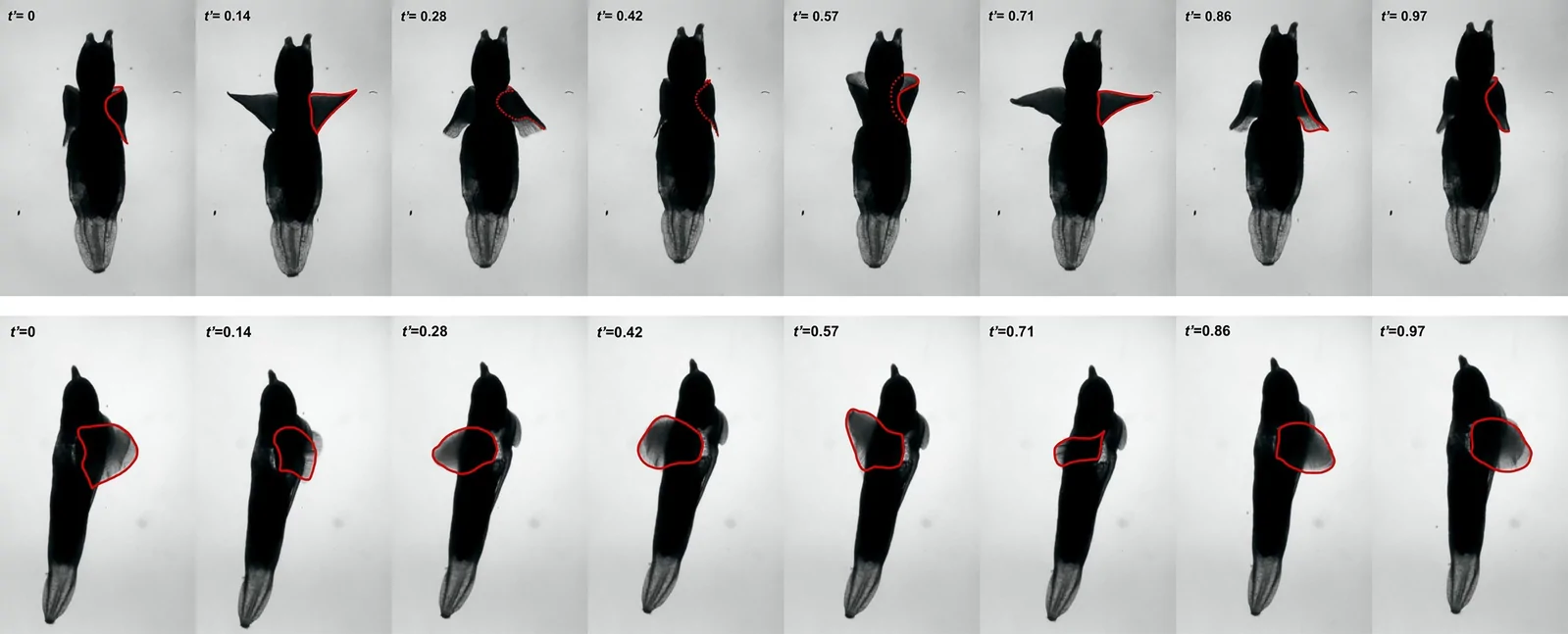
A sequence of images from two perpendicular synchronized high-speed cameras showing one complete cycle in hovering. The cycle starts at the middle of the recovery phase, denoted as *t’* = 0, where *t’* is the time normalized by cycle period. Each stroke cycle consists of two power and recovery phases. The right wing is outlined in red.

The photogrammetry system used here to measure the wing and body kinematics of *P. atlantica* was previously used by Karakas et al. (2020a) to measure the swimming kinematics of the thecosomatous pteropod *Cuvierina atlantica* and comprised two perpendicular high-speed Edgertronic cameras (1024 × 912 pixels; Sanstreak Corp., San Jose, CA, USA). The cameras were coupled to 200 mm Nikon macro lenses for high-magnification recording, with apertures set to f/32 to maximize the depth of field. The focal plane of each camera was set in the center of a glass aquarium (30 mm × 30 mm × 30 mm) filled with 0.2 µm filtered seawater to a depth of 28 mm to avoid filming animals interacting with the walls or the floor. The synchronized cameras, which had a spatial resolution of 14.3 µm pixel^−1^, filmed at 600 Hz. An LED fiber optic illuminator with a dual arm gooseneck (Dolan-Jenner Industries, Lawrence, MA, USA) provided collimated backlighting for each camera. All components were mounted on optical rails and a breadboard. The direct linear transform technique was used to spatially calibrate the camera system before data collection began (Hedrick 2008; Abdel-Aziz and Karara 2015; Karakas et al. 2020a). The pteropod was gently transferred to the aquarium, and the cameras were triggered when the animal entered the field of view of both cameras. Sixteen videos of the pteropod, denoted Animal 1, were collected. Two videos were chosen for analysis because the animal was well-focused in both camera views, far from the walls and the water surface, and performing either hovering or upward swimming. Thus, a 0.725 s portion of a recording in which the pteropod hovered in the common field of view for three wing stroke cycles was selected for further processing and analysis (Fig. 1). In addition, a 0.657 s portion of a second video in which the pteropod swims upwards for slightly more than three wing stroke cycles was also processed and analyzed. Like Borrell et al. (2005), we define each “figure-of-eight” wing stroke cycle to comprise two power phases (in which the wingtip moves downwards) and two recovery phases (in which the wingtip is drawn close the body as it gains elevation in preparation for the next power phase).

**Fig. 2:**
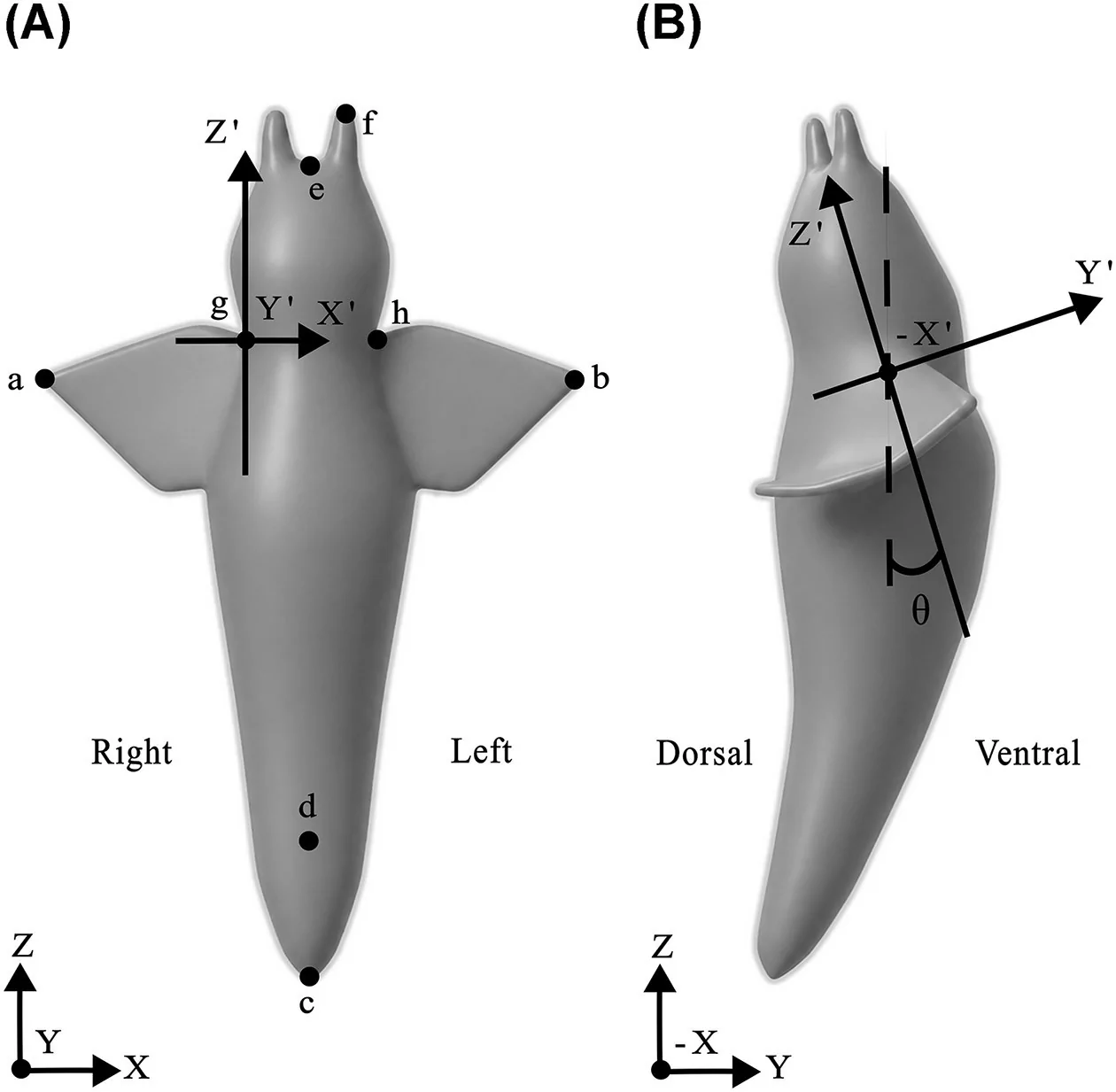
CAD model of sea angel showing the tracked points in body (X’Y’Z’) and global (XYZ) coordinate systems. As shown in (A), the body coordinate system, with point g as the origin, moved with the animal. The body angle θ was calculated by measuring the angle between Z and Z’ as shown in (B).

Eight different points on the pteropod (Fig. 2) were manually tracked in DLTdv8 (Hedrick 2008) to measure the 3D wing and body kinematics. Both a global coordinate system (XYZ) and a body-centered coordinate system (X′Y′Z′), which translates and rotates with the animal, were defined. The right wing base (point g) was defined as the origin of the body-centered coordinate system in order to enable comparison with mean wing kinematics data for the polar species *C. antarctica* recorded by Borrell et al. (2005; Borrell, personal communication). Further, we focused on the right wing because the left wing was partially occluded from one camera view during part of the stroke. Morphological parameters, including body length L_b_, calculated as the distance between the left buccal cone (point f) and tail (point c), wing length L_f_, calculated as the distance between the wingtip and wing base of the right and left wings (points a and g and b and h, respectively), and wingspan L_s_, calculated as the maximum distance between points a and b, were measured from the 3D kinematics data. In addition, the chord length L_c_ at the midpoint of the fully extended right wing was measured from the leading edge to the trailing edge using ImageJ. Wing lengths and chord lengths were measured when the wings were fully extended and averaged over multiple strokes to minimize error. For Animal 1, the projected area of the right wing was measured in one camera view when the wing was fully outstretched. The wing area *A* was then found by dividing the projected area by the sine of the wing angle with respect to the horizontal axis measured simultaneously in the orthogonal camera view. The mean chord length *\bar c* was calculated as $\bar c = {\raise0.7ex\hbox{$A$} {/ {\vphantom {A {{L_f}}}}} \!\lower0.7ex\hbox{${{L_f}}$}}$. The mean right wingtip speed u_wingtip_ was measured (in the body-centered coordinate system) for Animal 1 over three stroke cycles.

The beat frequency *f* was determined as the inverse of the average time required to complete one cycle. Instantaneous swimming speed *u* and average swimming speed *\bar u* also were calculated over three cycles based on point e. Body angle θ also was calculated as the angle between the Z axis and the Z’ axis (defined as a line connecting points e and d) such that positive values of θ correspond to the animal’s head pitching forward (ventrally). A body-based swimming Reynolds number was calculated as follows:


\begin{eqnarray*}
R{e_{\rm {swim}}} = \frac{{\bar u{L_b}}}{\nu }
\end{eqnarray*}

The wing-based chordwise Reynolds number was calculated as follows:


\begin{eqnarray*}
R{e_{\rm {chordwise}}} = \frac{{\bar c{u_{\rm {wingtip}}}}}{\nu }
\end{eqnarray*}

where *ν * is the kinematic viscosity of the seawater at 21°C (1.05 × 10^−6^ m^2^ s^−1^). The advance ratio J is also calculated as the ratio between the swimming speed and the average wingtip speed:


\begin{eqnarray*}
J = \frac{{\bar u}}{{{u_{\rm { wingtip}}}}}
\end{eqnarray*}

Finally, for Animal 1 (hovering) the stroke plane angles (β) were found by fitting a line to wingtip trajectory points projected onto the animal’s sagittal plane (in the body-centered coordinate system) during the two power phases of each of three stroke cycles and then calculating the respective angles relative to the horizontal (Y’) axis. The respective angles of attack (α) also were found by measuring the positions of the leading and trailing edges of the right parapodia at approximately mid-span and calculating the angle between this line (again projected onto the animal’s sagittal plane) and the respective stroke plane angle. For each power phase, measurements of α were taken at the middle portion of the stroke (eight time points centered at mid-stroke) and averaged. The stroke plane angles and average angles of attack were then measured for both the dorsal and ventral power phases of three stroke cycles to obtain average values. Though we have followed a similar procedure as others to measure α and β in pteropods (e.g., Borrell et al. 2005; Murphy et al. 2016), we note that these measurements are somewhat imprecise as the wings are highly flexible and bend substantially throughout the stroke.

### PIV setup

Flow generated by the swimming pteropod was quantified in two perpendicular, intersecting planes by the application of two time-synchronized, orthogonal, brightfield micro-PIV systems (Gemmell et al. 2014). The first micro-PIV system was similar to that used by Karakas et al. (2020a) and comprised a high-speed camera (Phantom VEO 640S) with a spatial resolution of 2560 × 1600 pixels which filmed at 700 Hz with a field of view of 12.43 × 7.77 mm^2^. A 2× extra-long working distance microscopic objective (no. 46–142, Mitutoyo) with tube lens (no. 58–520, Edmund Optics) generated a depth of field of approximately 91 µm and a measurement plane width (MPW) of 250 µm (Koutsiaris 2012). A collimated fiber optic illuminator (Dolan-Jenner) provided backlighting. The second micro-PIV system comprised a second high-speed camera (Phantom VEO 640S; 2560 × 1600 pixels), filming at 700 Hz and equipped with a 200 mm macro lens (Nikon) and two 27.5 mm extension rings to increase magnification to give a field of view of 18.29 × 11.43 mm^2^. Because the f-number of the lens was not recorded, the true depth of field cannot be calculated. However, assuming the maximum aperture (i.e., f/32) gives an estimate of the maximum depth of field of 784 µm (Wong et al. 2020). Illumination was provided by a fiber optic illuminator connected to a telecentric backlight illuminator (no. 62–760, Edmund Optics). The focal planes of both cameras were centered in the middle of the tank to film active swimming and avoid wall effects. *Nannochloropsis oculata* (mean diameter of 2–3 µm) were used as tracer particles. Two usable events in which a pteropod was present in the focal plane of both cameras were recorded by manual triggering. In the first video, the animal (denoted Animal 2) swims upwards at a slight angle. In the second video, a slightly faster animal (denoted Animal 3) turns while swimming horizontally.

The raw images were pre-processed using MATLAB (v9.14 R2023a, The MathWorks Inc., MA, USA). Median and Gaussian filters were applied to remove out-of-focus particles. To remove the animal from the image before PIV cross-correlation, an algorithmic mask was generated by implementing median and Gaussian filters and then binarizing the images. Dilation and erosion processes were done to minimize the black patches on the binarized mask of the animal. The resulting images were transferred to DaVis 10.2 PIV software (LaVision GmbH, Göttingen, Germany) for further processing and image cross-correlation to calculate flow fields. Subtraction of a sliding minimum and Gaussian smoothing were applied to reduce the variation in background illumination. Multipass cross-correlation (four initial passes with 75% overlap and 64 × 64 window size followed by another four passes with 50% overlap and 32 × 32 window size) between consecutive frames was used to calculate velocity vectors. Particle image displacements between consecutive frames did not exceed 8 pixels. Micro-PIV systems 1 and 2 had final vector spacings of 114.2 and 76.1 µm, respectively. For post-processing, the resulting vectors were denoised, and universal outlier detection was applied to remove spurious vectors. Uncertainty of the velocity magnitude averaged across each video sequence was estimated within the DaVis software as 6.2% for Animal 2 in micro-PIV system 1, 8.7% for Animal 2 in micro-PIV system 2, and 5.7% for Animal 3 in micro-PIV system 1. Finally, vorticity contours and velocity vectors were displayed on the original pteropod images using MATLAB.

Because the 3D wingtip kinematics could not be found for the PIV data, the chordwise Reynolds number and advance ratio were calculated as follows:


\begin{eqnarray*}
R{e_{\rm { chordwise}}} = \frac{{2\psi f{L_f}{L_c}}}{\nu }
\end{eqnarray*}

\begin{eqnarray*}
J = \frac{u}{{2\psi f{L_f}}}
\end{eqnarray*}

where *ψ * is the wing stroke amplitude (assumed as 160°) (Karakas et al. 2020a).

## Results

### Kinematic analysis

As shown in Table 1, Animal 1 had a body length of L_b_ = 7.9 mm, right and left wing lengths of L_R_ = 2.2 mm and L_L_ = 2.2 mm, respectively, and a chord length of L_c_ = 1.1 mm (and *\bar c* = 1.1 mm). This individual flapped its parapodia at 4.0 Hz and 4.9 Hz in hovering and upward swimming, respectively, to produce corresponding mean swimming speeds of 2 and 8.8 mm s^−1^. The corresponding swimming Reynolds numbers for hovering and upward swimming were $R{e_{\rm { swim}}}$ = 15 and 66, placing both swimming modes in the intermediate Reynolds number regime. In addition, this individual had $R{e_{\rm { chordwise}}}$ = 60 and 56 for hovering and upward swimming, respectively. Further, the advance ratio for hovering and upward swimming were J = 0.04 and 0.17, respectively. For Animal 2 (measured with the micro-PIV setup), the body length was longer (L_b_ = 10.1 mm), and the length of one wing was L_f_ = 2.3 mm, with chord length of L_c_ = 1.4 mm. Animal 2 flapped its appendages at 4.1 Hz and freely swam upward at 13.7 mm s^−1^ with advance ratio J = 0.26. The swimming and chordwise Reynolds numbers for Animal 2 were $R{e_{\rm { swim}}} = $ 136 and $R{e_{\rm { chordwise}}} = $ 87. For Animal 3 (measured with the micro-PIV setup), the body length was L_b_ = 9.2 mm, and the length of one wing was L_f_ = 2.2 mm, with chord length of L_c_ = 1.4 mm. Animal 3 flapped its appendages at 4.2 Hz to horizontally cross the field of view on one camera at 25.7 mm s^−1^, resulting in a higher advance ratio of J = 0.5. The corresponding swimming and chordwise Reynolds numbers were $R{e_{\rm { swim}}} = $ 232 and $R{e_{\rm { chordwise}}} = $ 85.

**Table 1:** Swimming characteristics of several gymnosome species. Values are given for angle of attack (α), stroke plane angle (β), body length (L_b_), wing length (L_f_), chord length (L_c_), swimming speed (*u*), wingbeat frequency (*f*), swimming Reynolds number (Re_swim_), chordwise Reynolds number (Re_chordwise_), and advance ratio (J). The angles of attack and stroke plane angles from Szymik and Satterlie (2011) are averaged across the two phases of the stroke cycle. With the exception of J (which represents free-swimming animals), the slow swimming data from Szymik and Satterlie (2011) combine tethered and free-swimming animals. The value of *L_c_* for Animal 1 in the current study is identical to the value of *\bar c*.

Species	Reference	α (deg)	β (deg)	L_b_ (mm)	L_f_ (mm)	L_c_ (mm)	*u* (mm s^−1^)	*f* (Hz)	Re_swim_	Re_chordwise_	J
*C. limacina* (hovering or slow swimming)	Satterlie et al. 1985	42.5	15–20	Up to 20	Can exceed 5	Up to 4.5	NA	1–3	NA	~200	NA
*C. limacina* (slow swimming)	Szymik and Satterlie 2011	61.6	11.1	NA	3.35–7.66	3.29–7.78	NA	1.79 ± 0.4	NA	152 ± 69	0.16–0.7
*C. limacina* (fast swimming)	Szymik and Satterlie 2011	66.6	16.3	NA	6.49–7.66	3.29–7.78	NA	2.80 ± 0.3	NA	201 ± 131	NA (tethered)
*C. antarctica*	Borrell et al. 2005	71.4 ± 2.2	24.8 ± 1.7	7–21	2.30–4.73	NA	1–7	0.81–1.62	6–49	60.8	0.06–0.18
*P. atlantica*	Karakas et al. 2020b	NA	NA	12.6	5.1 (wing span)	NA	18	4.0	224	NA	NA
*P. atlantica* - Animal 1 (hovering)	Current work	71.6 ± 10	35.4 ± 5.8	7.9	2.2	1.1	2.0	4.0	15	60	0.04
*P. atlantica* - Animal 1 (upward swimming)	Current work	NA	NA	7.9	2.2	1.1	8.8	4.9	66	56	0.17
*P. atlantica* - Animal 2	Current work	NA	NA	10.1	2.3	1.4	13.7	4.1	136	87	0.26
*P. atlantica* - Animal 3	Current work	NA	NA	9.2	2.2	1.4	25.7	4.2	232	85	0.5

**Fig. 3:**
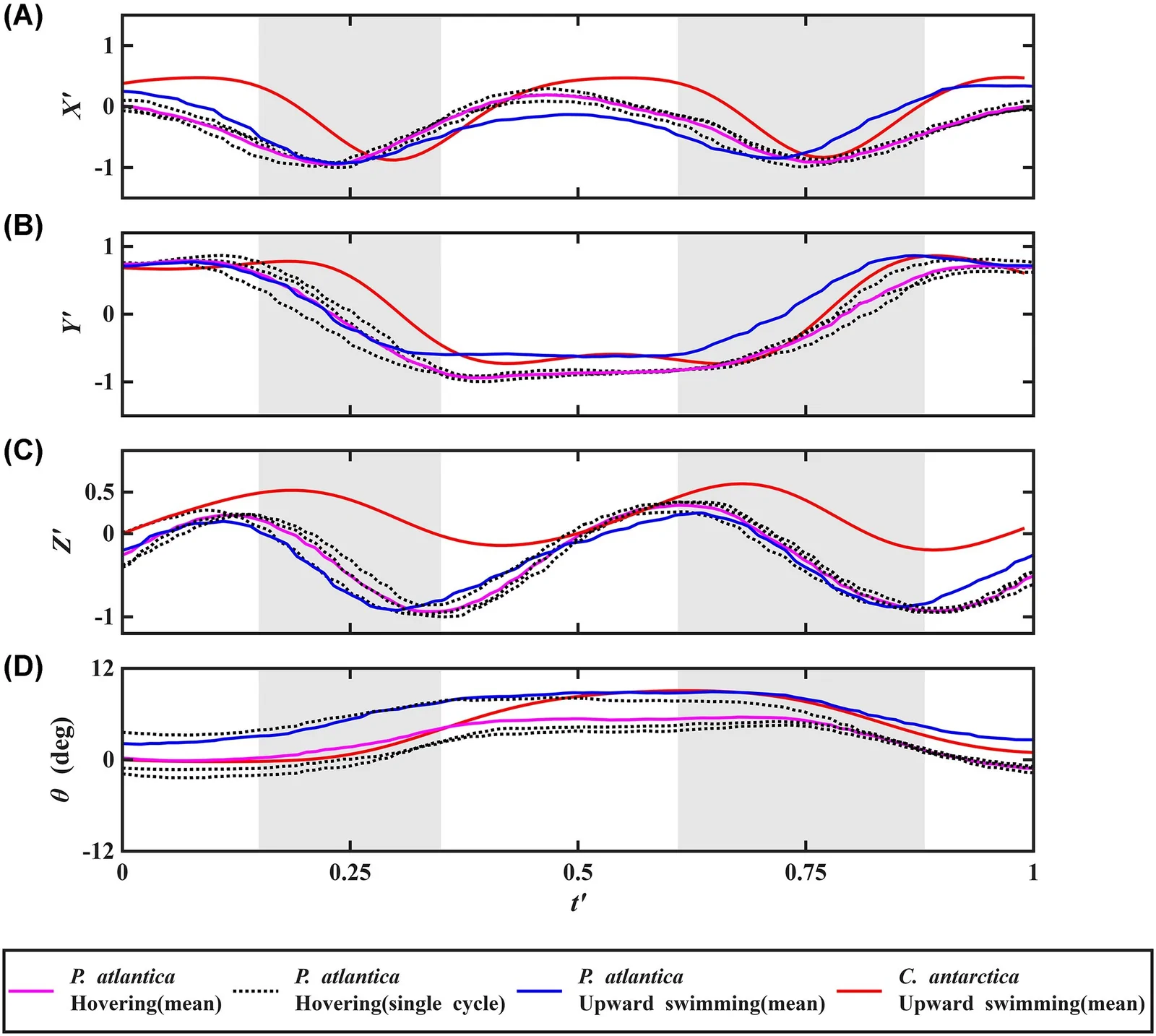
Kinematics of the right wingtip of *P. atlantica* in hovering and upward swimming. Wingtip positions are presented in the body-coordinate system and are normalized by the right wing length. Three consecutive cycles (black) and the mean cycle (magenta) are presented for hovering, and the mean cycle (blue) are presented for upward swimming. The mean kinematics for *C. antarctica* are presented for comparison (red; Borrell et al. 2005). As in Fig. 1, the cycle starts at the middle of the recovery phase, denoted as *t’* = 0, where *t’* is the time normalized by cycle period. The shaded and unshaded regions represent the power and recovery phases, respectively.

**Fig. 4:**
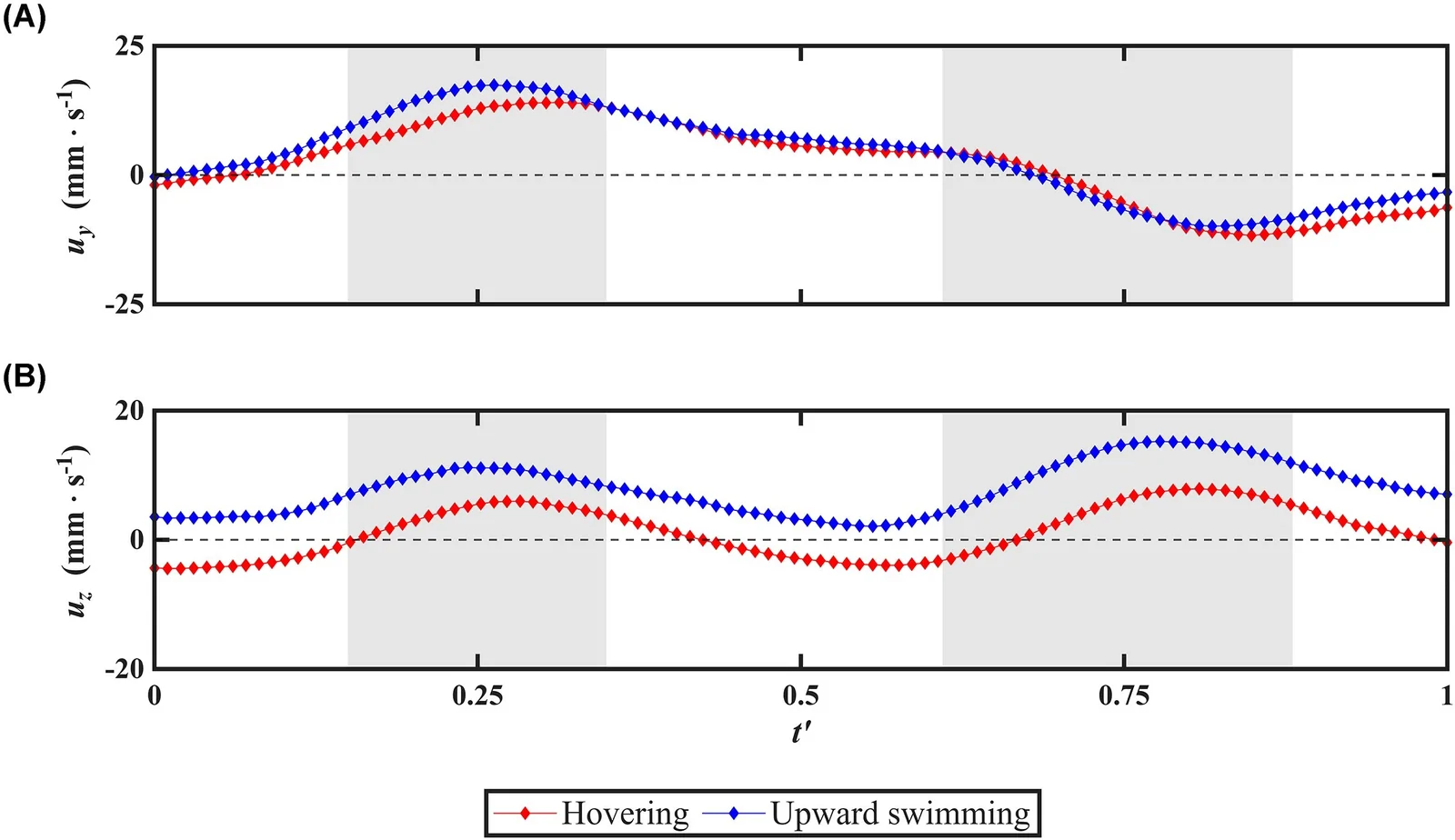
Average horizontal (A) and vertical (B) velocity of pteropod’s head (point e). Both velocities were normalized over three cycles (*t’*) for upward swimming and hovering modes. The shaded and unshaded regions represent the power and recovery strokes, respectively.

The wing and body kinematics of hovering *P. atlantica* (Animal 1) are described first, followed by a description of the kinematics of upwards swimming in the same animal. Figure 1 shows a complete cycle of Animal 1 hovering from two orthogonal views. Figure 3 shows the variation of the right wingtip position (in the body-centered coordinate system) and the body angle over time for both hovering (both mean and three individual cycles) and upwards swimming (mean). Mean wingtip positional data for upward swimming *C. antarctica* (Borrell et al. 2005) also are presented for comparison and are described later. Figure 4 shows the corresponding time variation of the average horizontal (i.e., dorso-ventral) body speed u_y_ and average vertical body speed u_z_. In these figures, *t’* is the time normalized by the period of one complete stroke cycle. Following Borrell et al. (2005), we define each half-stroke period to contain distinct power and recovery phases. The power phases of each stroke are shaded in Figs. 3 and 4.

For hovering, at *t’* = 0 the wings are in the middle of the recovery phase and are wrapped against the ventral side of the body as they begin to translate upwards (increasing Z’) to prepare for the next power phase. The wingtips do not overlap (Fig. 1). By *t’* = 0.14, the wings are initiating the power phase in which the wings move dorsally (decreasing Y’) and downwards (decreasing Z’) to generate lift. In reaction, the animal’s vertical speed increases to 6 mm s^−1^ and its body angle θ begins to increase as the animal pitches ventrally (Fig. 4B). At *t’* = 0.28, the power phase is nearing completion as the wingtips reach near their minimum in Z’ and are now moving medially (increasing X’) as they begin to wrap around the animal’s dorsal side. The second recovery phase is evident at *t’* = 0.42 as the wings, now pressed against the body, move upwards (increasing Z’) to prepare for the next power phase. During the recovery phase, the animal sinks slightly (Fig. 4B). At *t’* = 0.61, the pteropod begins its next power phase, and the wings move ventrally (increasing Y’) and downwards (decreasing Z’) as they sweep outwards away from the body (decreasing X’). Again, in reaction, the body pitches dorsally and its vertical speed increases to 8 mm s^−1^. The magnitude of the pitching angle during the entire stroke is approximately 5.6°. To complete its stroke (*t’* = 0.97), the wings again wrap closely to the body as they move upwards and prepare for the subsequent stroke. We note that the wings exhibit extensive spanwise and chordwise bending as they execute these complex maneuvers.

**Fig. 5:**
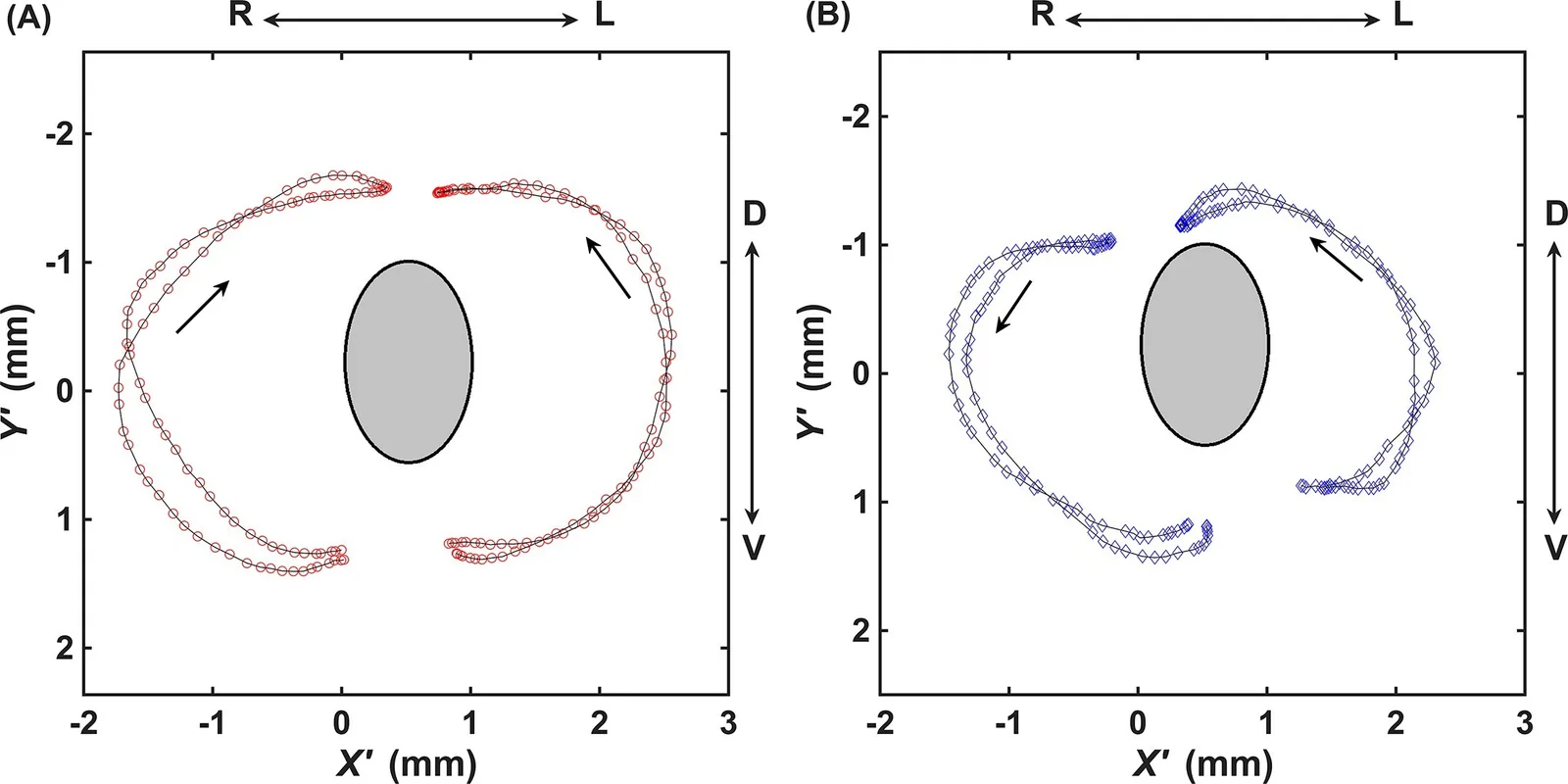
Mean right and left wingtip trajectories projected into the transverse plane for (A) hovering and (B) upwards swimming. The arrows indicate the wingtip direction. The right wing base is the origin of the coordinate system, and the gray ellipse represents the cross-section of the pteropod body in the transverse plane coinciding with the wing base location. “D” and “V” indicate the dorsal and ventral directions, respectively. “R” and “L” indicate the right and left directions, respectively.

Figure 5A shows the left and right mean wingtip trajectories for hovering as viewed from above the animal. In this figure, the animal’s body is represented as an ellipse, with the animal’s ventral side in the positive Y direction. The shape and size of the ellipse accurately represent the body shape of *P. atlantica* which is extended in the dorso-lateral direction, with the major and minor axes measured as 1.45 and 1.03 mm, respectively. Further, the wing attachment points (g and h) are ventrally offset from this minor axis by a distance of approximately 0.25 mm. Ventrally offset wing attachment points were also noted for *C. limacina*, though the body cross section at the wing attachment points is extended laterally instead of dorso-ventrally (Satterlie et al. 1985). We note that the right wingtip trajectories are likely more accurate because the left wingtip was occluded in one of the camera views during part of the two power phases. The description below is thus based on the right wingtip trajectory. The right wingtip traces out a half-circle pattern where it takes a slightly different trajectory during the ventral and dorsal portions of the two power stroke phases. Specifically, the wingtip extends further out (i.e., dorso-ventrally and laterally) during the first part of each of the two power phases, a pattern also seen in *C. limacina* (Satterlie et al. 1985; Szymik and Satterlie 2011).

**Fig. 6:**
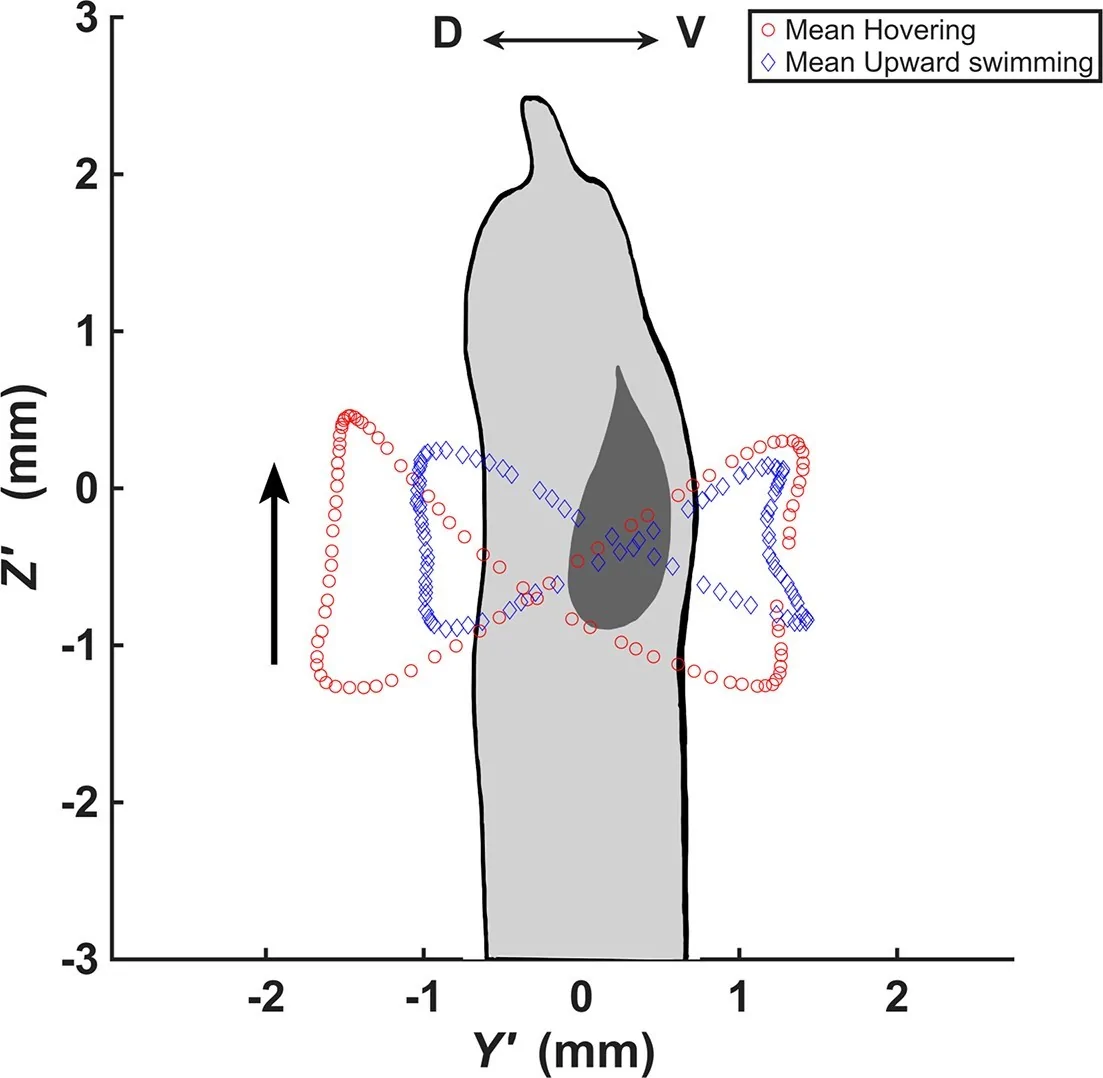
Right mean wingtip trajectories projected into the sagittal plane for hovering and upward swimming. “D” and “V” indicate the dorsal and ventral directions, respectively. The arrow indicates the wingtip’s direction of travel.

Figure 6 shows the average right wingtip trajectory for hovering Animal 1 projected into the sagittal plane. The wingtip traces out a quite symmetric figure-of-eight in which each half of the pattern resembles a triangle. The mean stroke plane angles in the dorsal and ventral power phases were 35.5° ± 1.6° and 35.3° ± 9.9°, respectively, and the corresponding angles of attack were 70.6° ± 9.5° and 72.7° ± 10.5°. Stroke plane angles and angles of attack for all six individual phases (i.e., combining dorsal and ventral power phases) were averaged to produce the α and β values presented in Table 1.

Animal 1 exhibited somewhat different kinematics while swimming upwards. Some of these differences may owe to the fact that the body of Animal 1 slightly rotated around a vertical axis as it swam upwards. For example, as seen in Fig. 5B, wingtip trajectories are rotated around the body’s long axis such that, on the body’s dorsal side, the right wingtip positions increase in the Y’ direction (i.e., move towards the body; Fig. 3B and Fig. 6) and increase in the X’ direction (i.e., move away from the body’s midline; Fig. 3A and Fig. 5B). In addition, the vertical extent of the right wingtip trajectory is somewhat less than that seen during hovering (Fig. 3C and Fig. 6). The pteropod thus seems to simply increase its beat frequency (from 4.0 to 4.9 Hz) rather than drastically alter its wing trajectories in order to increase its average speed (from 2.0 to 8 mm s^−1^). It should be noted that the corresponding increase in advance ratio is quite small (from 0.04 to 0.17), so it is perhaps not surprising that the wing kinematics remain quite similar. Indeed, the patterns in body oscillation (Figs. 3D and 4A) and in the vertical velocity (Fig. 4B) are very similar to hovering, but the pattern for vertical velocity is simply shifted upwards to a greater magnitude for upwards swimming.

### PIV results

**Fig. 7:**
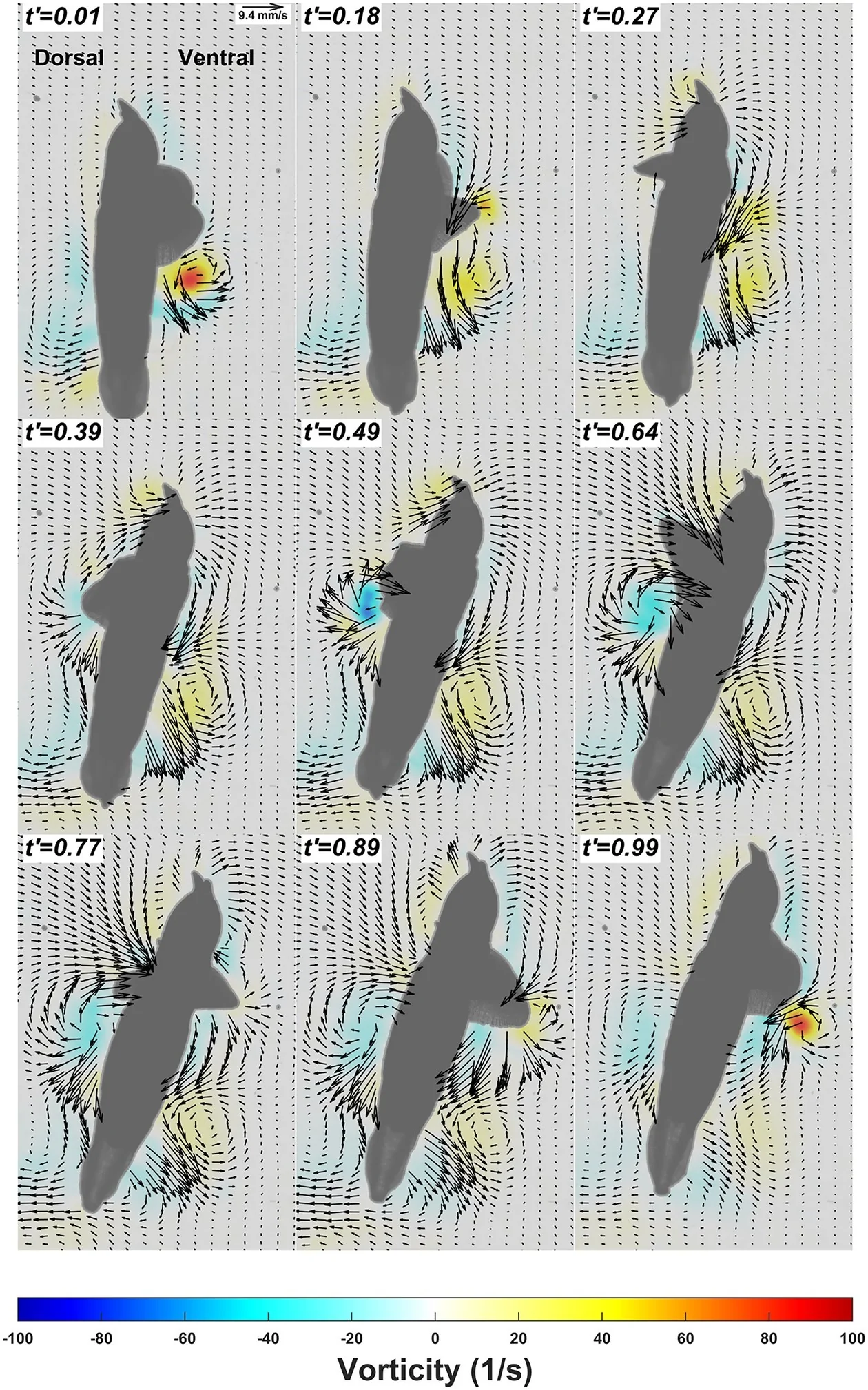
A sequence of images of Animal 2 from micro-PIV system 2 starting at the middle of the recovery stroke (*t’* = 0, where *t’* is the time normalized by cycle period). Color contours represent vorticity; the counterclockwise vortices are warm-colored, and clockwise vortices are cool-colored. Vectors indicate the flow direction and magnitude.

**Fig. 8:**
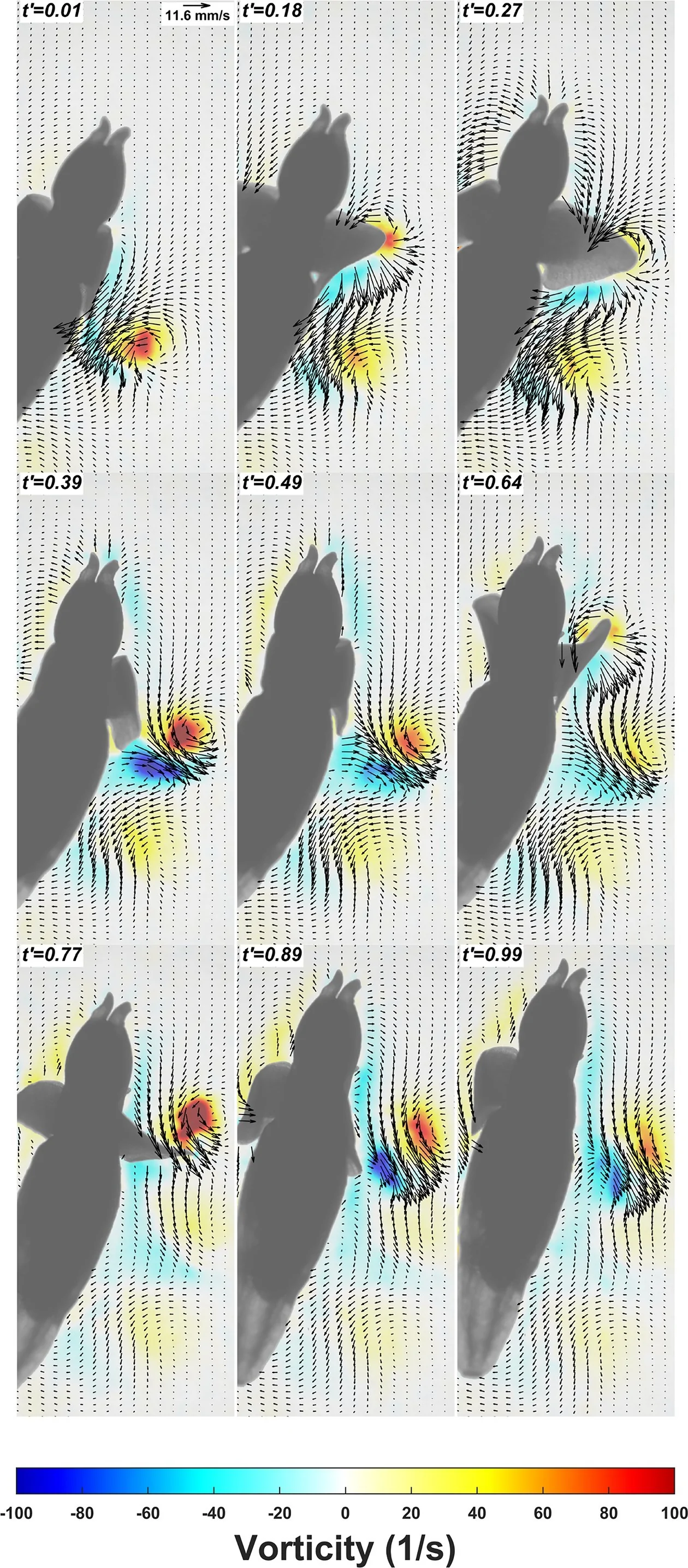
A sequence of images of Animal 2 from micro-PIV system 1 starting at the middle of the recovery stroke (*t’* = 0, where *t’* is the time normalized by cycle period). Color contours represent vorticity; the counterclockwise vortices are warm-colored, and clockwise vortices are cool-colored. Vectors indicate the flow direction and magnitude.

Figures 7 and 8 show a time sequence of flow fields for Animal 2 taken from the second and first micro-PIV systems, respectively. Movie 1 (in SI) shows the corresponding time sequence for both micro-PIV systems. In these figures, the vectors represent velocity and the color contours represent vorticity. Also, *t’* is again the time normalized by the stroke period of one complete cycle, where *t’* = 0 represents the middle of the ventral recovery phase. At this point (*t’* = 0.01 in Figs. 7 and 8), the wings are compressing against the body, which results in a downwards jet (with magnitudes of 25 mm s^−1^) squeezed from their trailing edge which then rolls up into what seems to be a vortex ring. The orthogonal view at the same time point (Fig. 8 and Movie 1) shows a strong downward flow (with magnitudes of 24.7 mm s^−1^) and a tip vortex from the wing previously sweeping through that region. By *t’* = 0.18, the wings are opening into a dorsally-directed power phase, and a strong downward flow (with magnitudes of 24.1 mm s^−1^) is seen between the wings and the body, thus providing evidence for the wing-body interactions posited by previous investigators (Satterlie et al. 1985; Szymik and Satterlie 2011). At *t’* = 0.27, this downwards flow continues to develop and merges with the jet previously squeezed downwards between the wings and body. By *t’* = 0.39, the wings are completing the power phase and, as they approach the body, they again squeeze flow downwards. The leading edges of the wings seem to approach the body before the trailing edges (in a cone-like configuration), thus contributing to the jet formation, a mechanism which mirrors the “cylindrical clap-and-fling” mechanism found by Karakas et al. (2020a) for *C. atlantica*. In addition, Fig. 8 and Movie 1 show a strong tip vortex forming lateral to the animal as the wingtip sweeps through the focal plane. By *t’* = 0.49, the squeeze flow induced by the wing-body interaction has rolled up into a vortex and continues to strengthen at *t’* = 0.64, with flow magnitudes up to 10.5 mm s^−1^ (Fig. 7). Further, at *t’* = 0.64, the wings have begun their next power phase, with flow moving down into a putative low-pressure region being created as the wings separate from the body. By *t’* = 0.77, a counter-rotating tip vortex is generated lateral to the body as the wing sweeps back through the focal plane of the first PIV system (Fig. 8 and Movie 1). At the last two time points (*t’* = 0.89 and 0.99), the wings again approach the body on the ventral side and the process repeats. We note that the squeeze flow is less apparent at *t’* = 0.99 but develops slightly later during the next stroke (Fig. 8 and Movie 1), which may be because the animal has moved slightly out of the PIV plane.

**Fig. 9:**
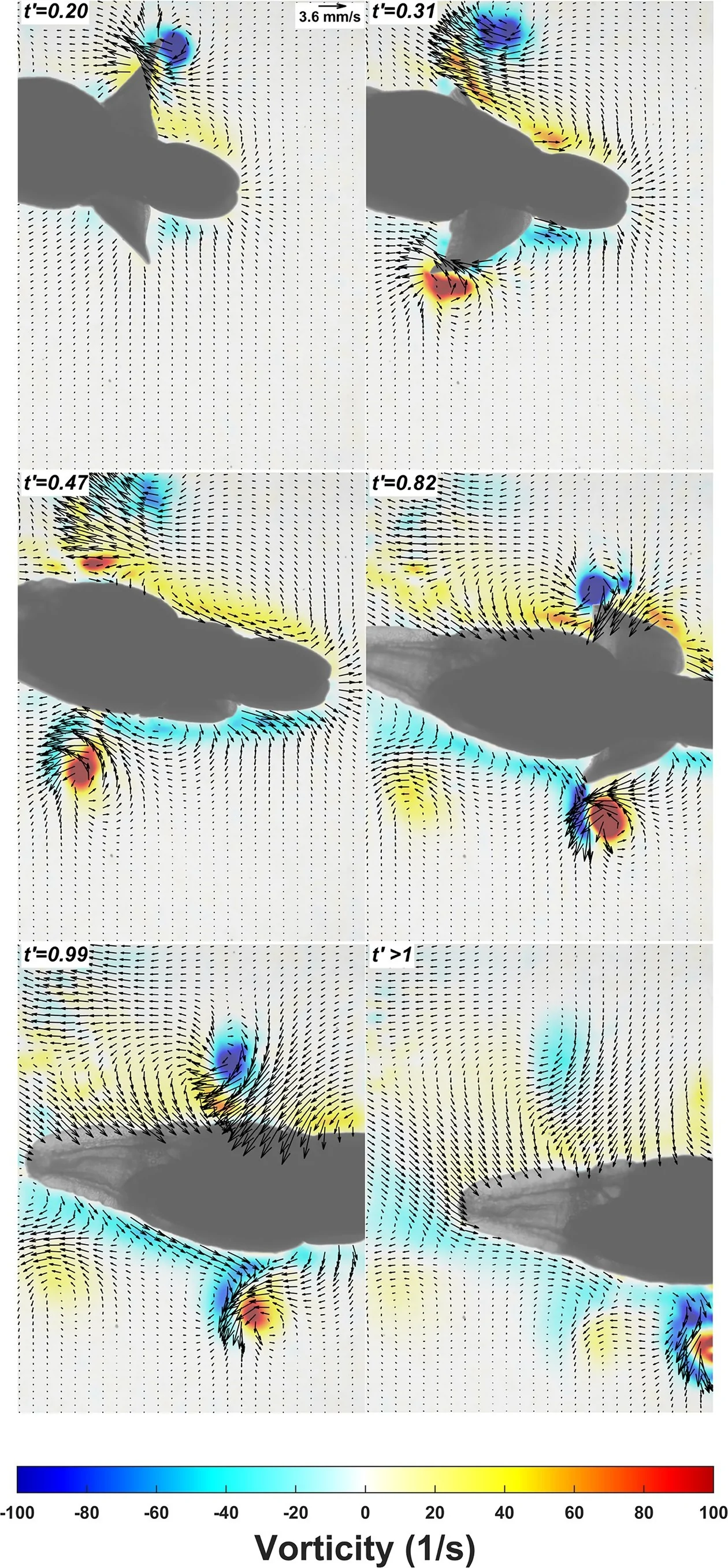
A sequence of images of Animal 3 from micro-PIV system 1. Color contours represent vorticity; the counterclockwise vortices are warm-colored, and clockwise vortices are cool-colored. Vectors indicate the flow direction and magnitude.

Figure 9 shows a time sequence of flow fields for Animal 3 taken from the first micro-PIV system. Movie 2 (in SI) shows the corresponding time sequence for both micro-PIV systems. As the animal is turning throughout the entire sequence (as is apparent in the view from the second micro-PIV system in Movie 2) and swimming sideways through the field of view, we base our description of the flow only on the first micro-PIV system. At *t’* = 0.20, the animal is in the middle of its power phase (though it is not possible to differentiate which phase is dorsally or ventrally directed). At this time point and at *t’* = 0.31, strong tip vortices are apparent on both wings, with velocity magnitudes up to 47 mm s^−1^. By *t’* = 0.47, the wings are wrapped close to the body. No evidence of wing-body interaction is seen from the flow measurements because the body occludes this region, but it is apparent that the wingtips overlap (Movie 2), indicating a stronger power phase and the possibility of hydrodynamically beneficial wing-body and wing-wing interactions. The vortices have grown somewhat larger and have moved downstream (i.e., opposite to the animal’s swimming direction). By *t’* = 0.82, the wings are most of the way through their next power phase and have again formed strong tip vortices, with magnitudes close to those formed in the previous power phase. At *t’* = 0.99 and beyond, the vortices continue to move opposite the animals’ swimming direction and to dissipate. In the last panel (*t’*>1), an additional tip vortex can be seen from the subsequent power phase on one side of the animal.

## Discussion

We have presented kinematics and flow measurements of *P. atlantica*, a rare warm-water gymnosome, performing various swimming behaviors. In particular, we quantified wing kinematics for hovering (J = 0.04) and slow upwards swimming (J = 0.17) in one individual (Animal 1) and provided the first flow measurements of a swimming gymnosome at somewhat higher advance ratios of J = 0.26 and 0.5 in two additional animals (Animals 2–3). Although the sample sizes are small for this rare species, these measurements allow us to perform an exploratory comparison of our results with the swimming of other gymnosome and thecosome species, examine the effects of swimming speed, and provide initial insight into the effect of Reynolds number on underwater flapping locomotion and into the role of wing-body and wing-wing interactions in gymnosome swimming.

**Fig. 10:**
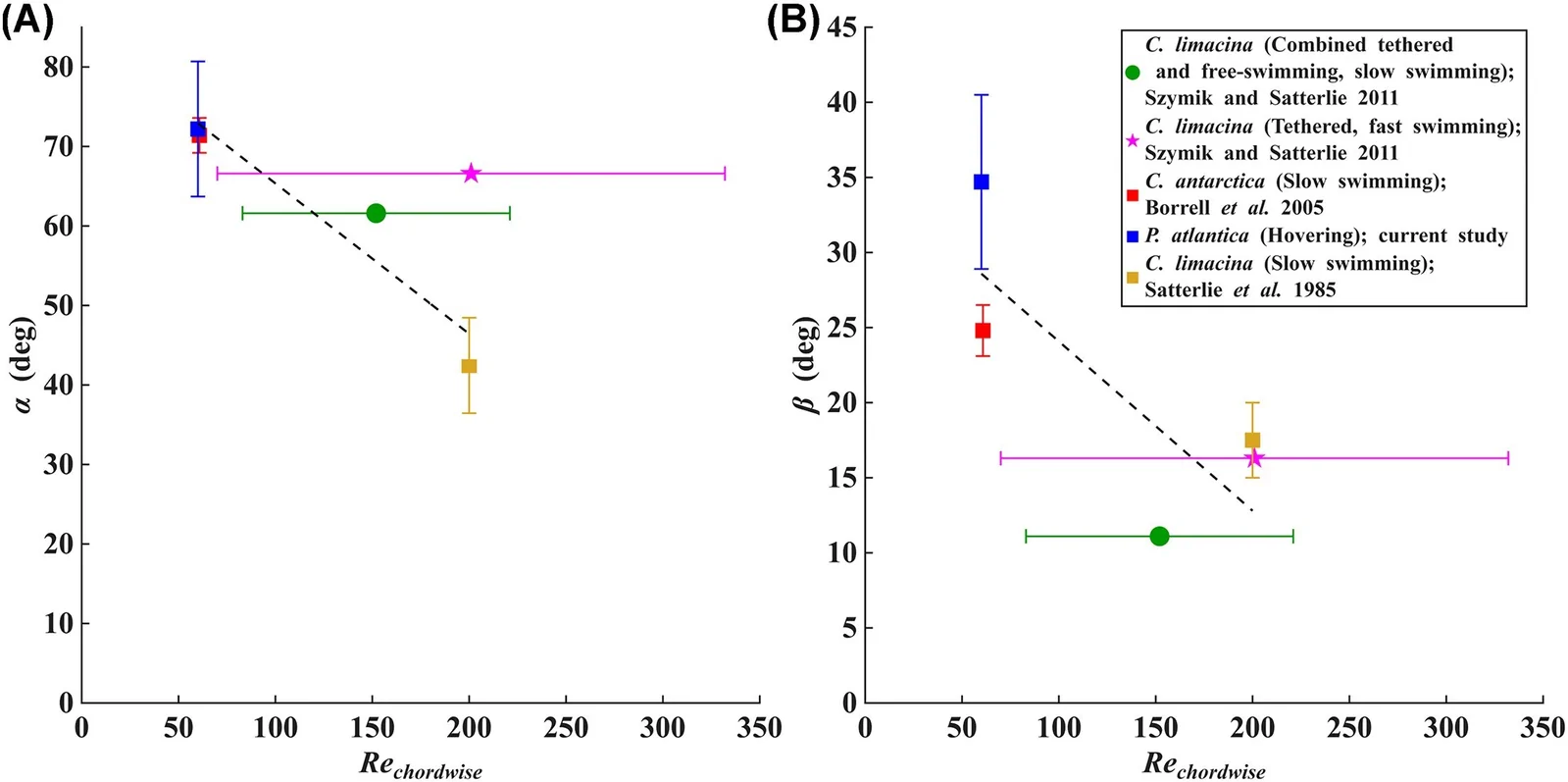
The relationship between chordwise Reynold number and (A) angle of attack α and (B) stroke plane angle β for three gymnosome species swimming in seawater at various temperatures. The error bars represent standard deviations for all data points except those of β for *C. limacina* (Satterlie et al. 1985), for which they represent a range. Regression lines are fitted to the four points representing hovering and slow swimming and are given by α = −0.18$R{e_{\rm { chordwise}}}$+83.8 (R^2^ = 0.90) and β = −0.12$R{e_{\rm { chordwise}}}$+35.8 (*R*^2^ = 0.59).

*Pneumoderma atlantica* exhibits largely similar wing kinematics as those observed in the temperate species *C. limacina* and polar species *C. antarctica*. However, several key differences emerge as a function of the Reynolds number at which these different species operate. First, as shown in Table 1, *P. atlantica* is somewhat smaller than the temperate or polar species and beats its parapodia at a correspondingly higher rate. In addition, the water viscosity is larger in temperate and polar waters than in subtropical waters. Specifically, *C. antarctica* swimming at temperatures of −2 to 2°C would experience a seawater kinematic viscosity in the range of ν = 1.72 × 10^−6^ to 1.95 × 10^−6^ m^2^ s^−1^, whereas *P. atlantica* experiences a kinematic viscosity of ν = 1.05 × 10^−6^ m^2^ s^−1^. However, the chordwise Reynolds numbers at which these different species operate are almost identical (approximately $R{e_{\rm { chordwise}}}$=~60) because the smaller size of *P. atlantica* is compensated for by its higher wingbeat frequency (Table 1). (We also note that the wing kinematics of these two species are highly similar, as seen in Fig. 3.) In contrast, *C. limacina*, which are large and swim at intermediate viscosities, experience the highest chordwise Reynolds numbers ($R{e_{\rm { chordwise}}}$=~150–200). These differences in wing Reynolds number mean that *P. atlantica* and *C. antarctica* experience higher viscous forces than *C. limacina*. This greater importance of viscous forces may possibly translate into corresponding differences in the stroke plane angle and angle of attack for animals operating at lower Reynolds numbers. Specifically, Figs. 10A and 10B show plots of the chordwise Reynolds number versus the angle of attack (α) and power phase stroke plane angle (β), respectively. Figures 10A and 10B also show lines fit to the four hovering and slow-swimming data points for freely swimming animals, though we note that the slow-swimming data from Szymik and Satterlie (2011) include both tethered and free-swimming animals. We have excluded the fast-swimming tethered data from Szymik and Satterlie (2011) from this regression as both tethering and fast swimming are likely to alter the wing and body kinematics. As seen in both Fig. 10A and 10B, higher Reynolds numbers appear to correspond to lower angles of attack and lower stroke plane angles. We note that caution is warranted in interpreting these relationships owing to the small number of data points. However, a similar relationship between wing Reynolds number and angle of attack and stroke-plane angle is also seen in tiny insects operating in a similar Reynolds number range. For example, Lyu et al. (2019) found that the stroke plane of tiny insects increased as the wing Reynolds number decreased from several hundred to approximately ten (e.g., Williams et al. 2025). The gymnosomes *P. atlantica* and *C. antarctica* and these tiny insects, all operating in a similar low Reynolds numbers regime, thus appear to rely more heavily on drag forces on their wings to generate upwards lift. We do note that various authors have defined $R{e_{\rm { chordwise}}}$ differently, which may slightly affect the results presented in Fig. 10. For example, Szymik and Satterlie (2011) used the peak wingtip speed whereas we and Borrell et al. (2005) use the mean wingtip speed. The method by which Satterlie et al. (1985) calculated $R{e_{\rm { chordwise}}}$ is not given. Nonetheless, these differences are likely not large enough to affect the general trends in Figs. 10A and 10B. We also note that species-specific differences in wing planform, wing flexibility, and body cross-sectional shape may affect wing kinematics.

Both tiny insects and gymnosomes also use unsteady flow mechanisms to generate additional lift (Ellington 1984). Tiny insects generate approximately 30% of their lift using the Weis-Fogh “clap-and-fling” mechanism (Cheng and Sun 2018), and gymnosomes seem to use a wing-body interaction to generate additional lift. Indeed, the flow measurements (Figs. 7 and 8) show flow moving down into the presumed low-pressure gap between the wings and body and being squeezed out from this region beneath the wings. Karakas et al. (2020a) found a similar mechanism for the similarly sized *C. atlantica*, and recent flow simulations of this species have shown that the wing–wing interaction in this cylindrical overlap and fling mechanism enhances lift by approximately 20% as compared to a single-wing case with no such wing-wing interaction (Li et al. 2026). Similar simulations are needed to gauge the importance of the wing-body interactions in gymnosomes. In gymnosomes, the amount of wingtip overlapping may correspond to the importance of such wing-body interactions in force generation. For example, the fast-swimming gymnosome in Fig. 9 clearly exhibits a greater amount of wingtip overlapping than the hovering animals, where overlapping is essentially absent. In addition, Szymik and Satterlie (2011) showed that the wingtips of tethered *C. limacina* approached more closely and overlapped more at the higher flapping frequencies corresponding to fast swimming. A greater amount of overlapping would minimize the enclosed volume, thus generating greater squeeze flow from the wings’ trailing edges and greater volume for a presumed low-pressure suction region to form. We also note that the temperate and polar species seem to exhibit greater wingtip overlapping than for *P. atlantica*. This difference may arise from the different morphology of these different species (in particular the medially compressed body of *P. atlantica* versus the dorsoventrally compressed body of the temperate and polar species) or from size and Reynolds number differences. We also note that the frontal lobe of *P. atlantica* (located on the ventral side; Fig. 2B) bulges out somewhat, which may affect the volume which the wings can create when they approach close to the body. The force generated by wing-body interactions may thus differ ventrally and dorsally. In a similar vein, the effect of the gap between wingtips on aerodynamic performance of insects was investigated before; a simulation of a tiny insect model by Miller and Peskin (2009) showed that minimizing this gap strengthens the vortices and enhances lift generation. Also, Lehmann et al. (2005) found using dynamically scaled *Drosophila* wings that the maximum lift augmentation (i.e., up to 3.9%) occurs when the wingtips are nearly touching. Future computational studies could similarly investigate the effect of altering stroke kinematics and wing-body interaction on lift enhancement in gymnosomes.

## Limitations

A significant limitation of this study is the low sample size (*n* = 3) of individual pteropods examined. This low number stemmed from the rarity of this species, which is a well-established trend for pteropods at low latitudes (Burridge et al. 2017), and limits the strength of our conclusions. Further, these pteropods do not live long after capture, which restricts experimental investigation. An additional limitation of our experimental setup was our inability to reconstruct the 3D wing and body kinematics from the two orthogonal micro-PIV systems. While these two systems did provide useful information about the flow in perpendicular planes, the depth of field was too narrow to allow accurate calibration. Our kinematics data are thus limited to slow swimming while the flow measurements are limited to somewhat faster swimming. Further, for Animal 1, one wingtip was occluded in one camera view during part of the wing stroke, making it difficult to gauge left-right symmetry. Nonetheless, this study provides new insight into the swimming hydrodynamics of subtropical pteropods and, like previous investigations of shelled pteropods (Chang and Yen 2012; Adhikari et al. 2016; Murphy et al. 2016; Karakas et al. 2020b) and heteropods (Karakas et al. 2018), highlights the similarities between the swimming of zooplanktonic marine snails and the flight of insects.

## Supplementary Material

icag160_Supplemental_Files

## Data Availability

All data are available upon reasonable request.
